# Prognostic significance of low microRNA-218 expression in patients with different types of cancer: Evidence from published studies

**DOI:** 10.1097/MD.0000000000004773

**Published:** 2016-09-16

**Authors:** Fujiao Duan, Kaijuan Wang, Liping Dai, Xia Zhao, Yajing Feng, Chunhua Song, Shuli Cui, Chengzeng Wang

**Affiliations:** a Department of Hospital Infection Management; b Department of Ultrasound, Affiliated Cancer Hospital of Zhengzhou University, Zhengzhou, Henan, China; c Department of Epidemiology, College of Public Health, Zhengzhou University; d Department of Nosocomial Infection Management, The First Affiliated Hospital of Zhengzhou University; e Henan Key Laboratory of Tumor Epidemiology, Zhengzhou, Henan, China; f College of Professional Study, Northeastern University, Boston, MA.

**Keywords:** cancer, miR-218, prognosis, quantitative evaluation

## Abstract

**Background:**

Mounting evidence showed that microRNAs may be useful as prognostic biomarkers of cancer. Therefore, we summarize the predictive role of microRNA-218 (miR-218) for survival in patients with various cancers.

**Methods:**

We performed a systematic literature review and assessed the quality of included studies based on Meta-analysis of Observational Studies in Epidemiology group (MOOSE). Hazard ratios (HRs) with corresponding 95% confidence intervals (CIs) were calculated to assess the correlation between miR-218 expression and prognosis of different cancers.

**Results:**

We identified 10 studies for pooled analyses. For overall survival, a lower expression levels of miR-218 significantly predicted poorer survival, with the pooled HR of 2.61 (95% CI: 2.11–3.22, *P* < 0.001). For disease-free survival/progressive-free survival/recurrence-free survival (DFS/PFS/RFS), a lower expression level of miR-218 significantly predicted worse DFS/PFS/RFS in various carcinomas, with the pooled HR of 2.73 (95% CI: 2.08–3.58, *P* < 0.001). Similarly, subgroup analysis by detection method, ethnicity and cancer subtype analysis suggested that lower expression of miR-218 correlated with.

**Conclusion:**

Our data demonstrated that lower miR-218 expression is significantly associated with poorer overall survival (OS) and DFS/PFS/RFS and may be a novel prognostic biomarker in some cancer types.

## 1 Introduction

Cancer is a major public health problem in the world.^[1]^ Although overall cancer mortality decreased by 20% between 1991 and 2010, cancer remains one of the most common causes of death worldwide.^[2]^ The prognosis in the most cancers remains unsatisfactory, especially for advanced-stage tumors. Tumor metastasis is a complex process and a major cause of cancer deaths.^[3]^ Therefore, it is necessary to identify valuable molecular biomarkers to promote early detection, prognostic classification, and novel therapeutic strategies for cancers.

MicroRNAs (miRNAs) are evolutionary conserved, small noncoding molecules with approximately 22 nucleotides in length, which could bind to complementary sequences in the 3′ untranslated region (3′UTR) of target mRNAs, leading to mRNA degradation or translational repression.^[4]^ They have been shown to regulate multiple biological processes such as cell proliferation, cell differentiation, cell apoptosis, and cell cycle regulation.^[5,6]^ Mounting evidence suggests that some miRNAs may function as oncogenes or tumor suppressors by regulating cell proliferation and other related biological behaviors.^[7,8]^

MicroRNA-218 (miR-218) belongs to the *silt* gene family, target recognition and regulatory functions as a onco-suppressor gene.^[9,10]^ Several studies have reported that miR-218 expression was significantly downregulated in cancer tissues and played a role in cancer progression.^[11,12]^ The role of miR-218 in the identification and characterization of tumor-initiating cells in cancers may provide new insight into understanding the relation of molecular mechanisms of tumor development.^[13]^ Therefore, the development of new therapy options is essential.

Recent studies showed that miRNAs are associated with prognosis in various carcinomas, suggesting that they could be developed as prognostic classifiers to guide therapeutic decisions. We performed the systematic review of the data available from studies published in this field with the main aim of evaluating the role of miR-218 as a prognostic biomarker in cancer.

## 2 Materials and methods

Ethics committee is not applicable in this meta-analysis.

The present study was performed in accordance with the guidelines of the Meta-analysis of Observational Studies in Epidemiology group (MOOSE) issued by Stroup et al^[14]^ and Preferred Reporting Items for Systematic Review and Meta-analyses (PRISMA) criteria.^[15]^

### 2.1 Literature search strategy

We systematically searched PubMed, Embase, Web of Science, Chinese National Knowledge Infrastructure (CNKI), and Wanfang database to identify potential studies before January 1, 2016. The search strategy employed terms related to “microRNA-218” or “miR-218” and “neoplasms” or “cancer.” The search was limited to papers published in English or Chinese language. In addition, reference lists of retrieved articles were examined manually to further identify missing relevant publications.

### 2.2 Inclusion and exclusion criteria

Two reviewers (FD and LD) independently assessed eligibility of the retrieved articles. Studies were included in the analysis if the following criteria were met: the study subjects were patients with any type of cancer; miR-218 expression was measured in tumor tissue or serum; investigated the survival outcome or the correlation between miR-218 expression and the clinical variables; and the full-text article was available in English or Chinese. Studies were excluded based on the following criteria: reviews, laboratory studies or letters; non-English or Chinese articles; lacked key information regarding survival outcomes, such as HRs or 95% confidence intervals (95% CIs) or unable to calculate such parameters.

### 2.3 Data extraction and quality assessment

Two investigators (FD and KW) evaluated and extracted the data independently from all eligible studies under the guideline of a critical review checklist. Data for analyses, including first author, year of publication, origin country, histology, sample type and size, assay, follow-up and cutoff value, HRs of miR-218 for overall survival (OS) and/or disease-free survival (DFS), progressive-free survival (PFS), recurrence-free survival (RFS), and the corresponding 95% CIs. If not available, data were calculated following Tierney et al's method.^[16]^ If discrepancies existed, consensus would be finally reached on discussion.

The methodological quality of each study was systematically assessed according to a critical review checklist of the Dutch Cochrane Centre proposed by MOOSE to ensure their quality.^[14]^ The key points of the basic standard are as follows: study origin of country and population, type of carcinoma, study design, outcome assessment, measurement of miR-218, cut-off of miR-218, and sufficient follow-up. The study was removed if not including the basic standard to avoid compromised quality of the meta-analysis.

### 2.4 Statistical analysis

We utilized RevMan 5.3 (Cochrane Collaboration, Oxford, UK) and STATA 13.1MP (StataCorp, College Station, TX) to perform all the statistical analysis.

All of the HRs and corresponding 95% CIs were used to calculate the pooled HR. Cochran *Q* test and Higgins *I*^2^ statistic were used to assess heterogeneity, if *P*-value for heterogeneity test (*P*_heterogeneity_) < 0.05 or *I*^2^ > 50%, the sources of heterogeneity would be used for meta-regression.^[17]^ Random or fixed-effects models were used depending on *P*_heterogeneity_. If *P*_heterogeneity_ ≥ 0.05, we used the fixed effect model (the Mantel–Haenszel method).^[18]^ Otherwise, random effects model (DerSimonian and Laird method) was selected.^[19]^ The significance of merged HR was dependent on the *Z* test, *P* < 0.05 was considered statistically significant, all *P* values were 2-sided.

Sensitivity analysis, in which 1 study is omitted at a time, was performed to assess the quality and consistency of the results.

Publication bias was assessed by Begg test (rank correlation test)^[20]^ and then statistically using Egger test (weighted linear regression test).^[21]^

## 3 Results

### 3.1 Literature search and summary of included studies

**Figure 1:**
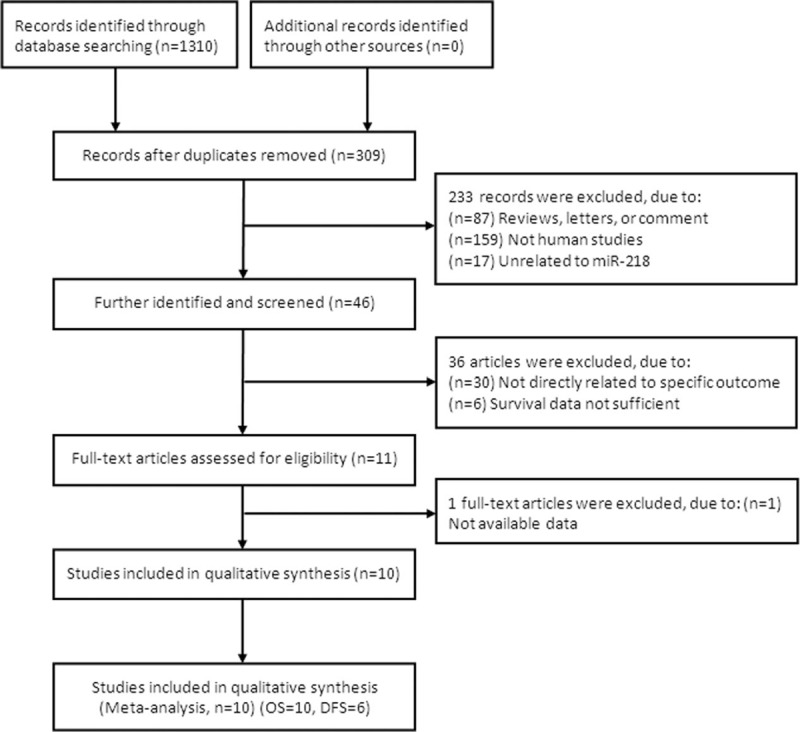
Flow chart of literature search and study selection.

The initial literature search retrieved 1310 relevant studies and a flow diagram are shown in Fig. 1. One thousand one studies were removed because of duplication. After primary identified, 46 titles were potentially appropriate, and the corresponding abstracts were reviewed. After further identification and screening individual study, 11 eligible publications underwent full-text review, and 1 article^[22]^ was further excluded because data were unavailable. Finally, we included 10 eligible studies^[12,13,23–30]^ in the final evidence synthesis.

The main characteristics of the eligible studies are summarized in Table 1. The eligible studies were published from 2010 to 2015 and included a total of 893 participants with OS data and 626 participants with DFS/PFS/RFS data from China, Taiwan, and Canada. The patients were classified as either Asian or Caucasian according to their ethnic background. The types of malignant cancers included colorectal cancer, nonsmall cell lung cancer (NSCLC), pancreatic cancer, oral cavity squamous cell carcinoma (OCSCC), nasopharyngeal carcinoma (NPC), glioma, and hepatocellular carcinoma (HCC). Frozen tissues or serum were used in eligible studies. Quantitative real-time PCR (qRT-PCR) was used in 8 studies, and immunohistochemical (IHC) was used in the remaining 2 studies.

**Table 1:**
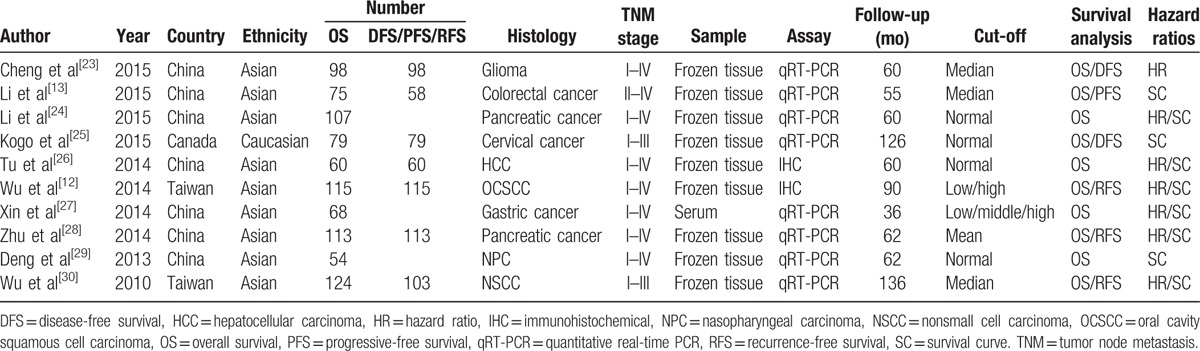
Clinicopathological characteristics of eligible studies.

Among the eligible studies, 10 articles^[12,13,23–30]^ evaluated both OS and DFS/PFS/RFS, 6 articles^[12,13,23,25,28,30]^ evaluated DFS/PFS/RFS. Seven studies^[12,23,24,26–28,30]^ directly reported HRs and 95% CIs, three studies^[13,25,29]^ reported survival curve (SC).

### 3.2 Evidence synthesis and test of heterogeneity

**Figure 2:**
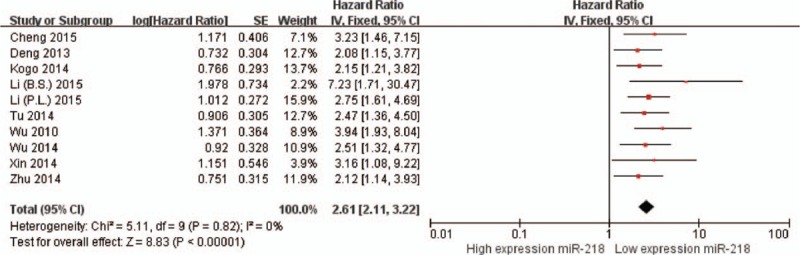
Forest plots of studies evaluating the HRs of high and low miR-218 expression with respect to OS. The squares and horizontal lines correspond to the study-specific OR and 95% CI. The area of the squares reflects the study-specific weight. The diamond represents the pooled OR and 95% CI.

**Figure 3:**
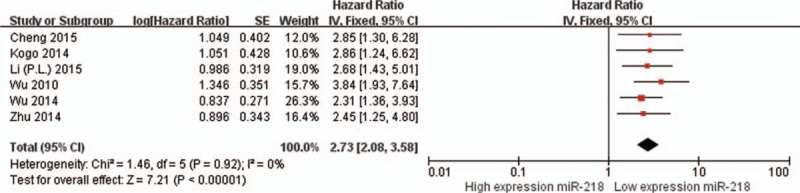
Forest plots of studies evaluating the HRs of high and low miR-218 expression with respect to DFS/PFS/RFS.

The main results of this meta-analysis and the heterogeneity test are shown in Table 2. We firstly analyzed the association between miR-218 expression and OS, no significant heterogeneity have been found (*I*^2^ < 0.001%, *P* = 0.82). Therefore, the fixed effects were applied to calculate the pooled HR, a lower expression levels of miR-218 significantly predicted poorer survival, with the pooled HR of 2.61 (95% CI: 2.11–3.22, *P* < 0.001, Fig. 2). For evaluating the association between miR-218 expression and DFS/PFS/RFS, since the *Q* test of heterogeneity was not significant (*I*^2^ < 0.001%, *P* = 0.83), we conducted analyses using the fixed effect models. The result showed that a lower expression level of miR-218 significantly predicted worse DFS/PFS/RFS in various carcinomas, with the pooled HR of 2.73 (95% CI: 2.08–3.58, *P* < 0.001, Fig. 3).

**Table 2:**
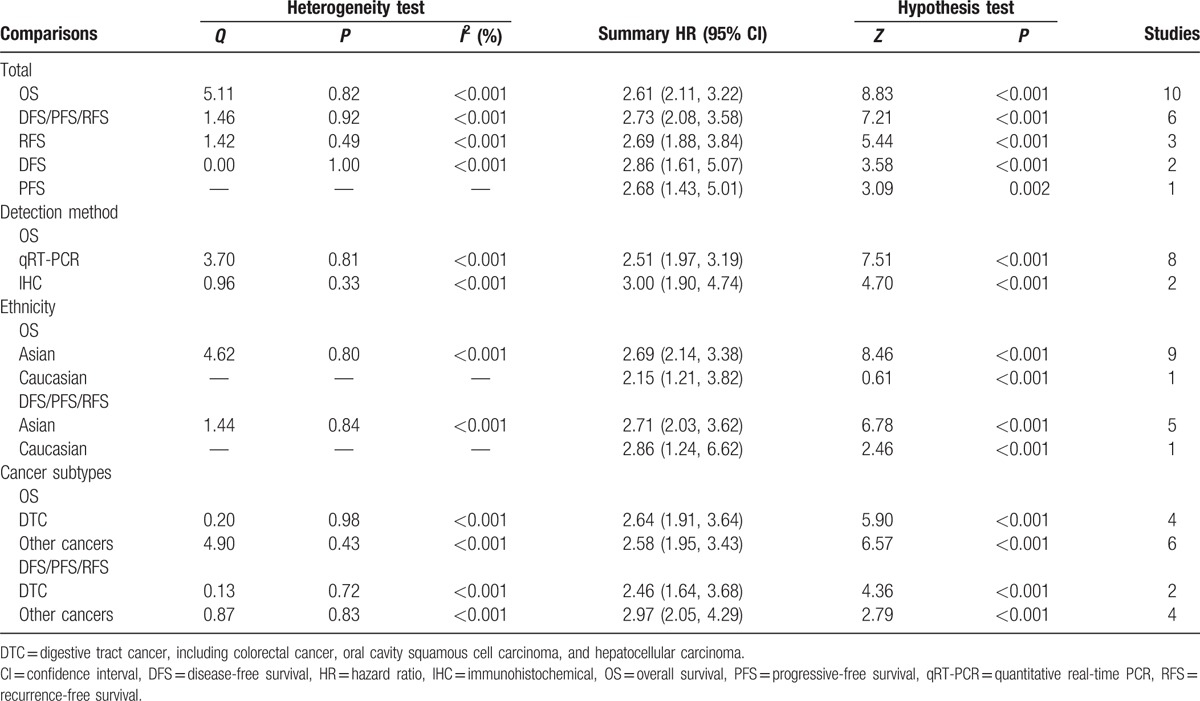
Main results of pooled HRs in the meta-analysis.

To explain the heterogeneity in OS, subgroup analysis was performed by detection method, significant relevance was observed both in qRT-PCR subgroup (HR = 2.51, 95% CI: 1.79–3.13, *P* < 0.001) and IHC subgroup (HR = 3.00, 95% CI: 1.90–4.74, *P* < 0.001). Considering the large proportion of Chinese patients in the studies, we carried out a stratified analysis by classifying studies into subgroups of ethnicity (Asian and Caucasian). The expression of miR-218 was significantly correlated with OS in Asians (HR = 2.69, 95% CI: 2.14–3.38, *P* < 0.001) and Caucasians (HR = 2.15, 95% CI: 1.21–3.82; *P* < 0.001) (Table 2) and expression of miR-218 significantly associated with DFS/PFS/RFS in Asians (HR = 2.71, 95% CI: 2.03–3.62, *P* < 0.001) and Caucasians (HR = 2.86, 95% CI: 1.24–6.62, *P* = 0.01) (Table 2). When grouped by the cancer types, we found that miR-218 expression was significantly correlated with digestive tract cancer (DTC) (HR = 2.64, 95% CI: 1.91–3.64, *P* < 0.001 for OS; HR = 2.46, 95% CI: 1.64–3.68, *P* < 0.001 for DFS/PFS/RFS) and other cancers groups (HR = 2.58, 95% CI: 1.95–3.43, *P* < 0.001 for OS; HR = 2.97, 95% CI: 2.05–4.29, *P* < 0.001 for DFS/PFS/RFS) (Table 2).

### 3.3 Sensitivity analysis

**Figure 4:**
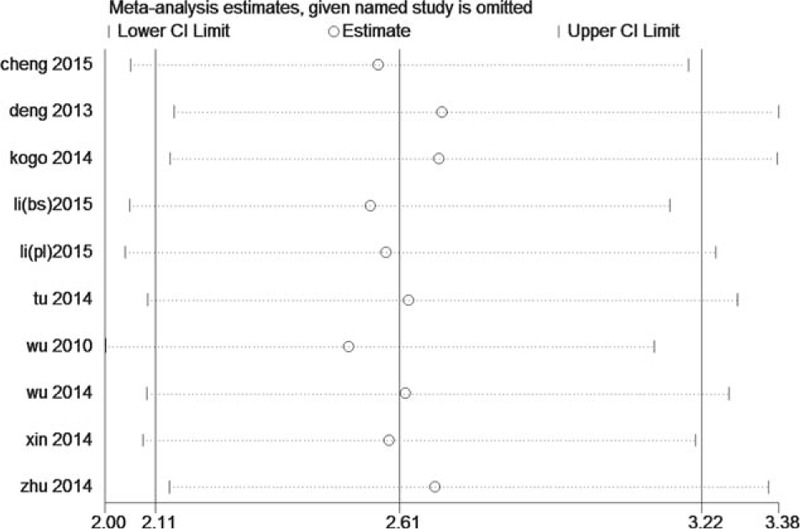
Sensitivity analysis for OS of miR-218.

**Figure 5:**
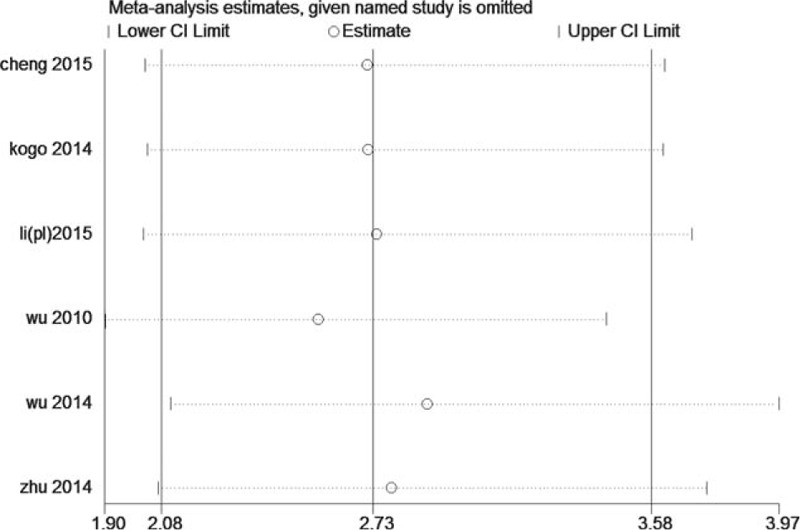
Sensitivity analysis for DFS/PFS/RFS of miR-218.

Sensitivity analysis was performed through systematic omitting 1 study each time and calculating the pooled HRs again. As shown in Figs. 4 and 5, the stability of the entire study was not influenced by 1 individual study.

### 3.4 Evaluation of publication bias

**Figure 6:**
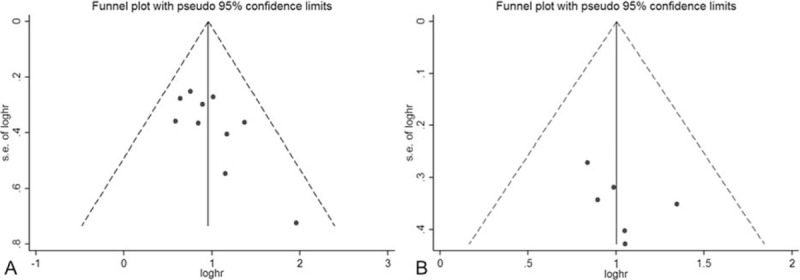
(A) Funnel plot of publication bias on the relationship between miR-218 expression and OS. The vertical line in the funnel plot indicates the fixed-effects summary estimate, whereas the sloping lines indicate the expected 95% CI for a given SE. (B) Funnel plot of publication bias on the relationship between miR-218 expression and DFS/PFS/RFS.

Begg funnel plot and Egger linear regression test were performed to assess the publication biases of OS and DFS/PFS/RFS among included studies. The shape of the funnel plot did not reveal any evidence of obvious asymmetry (Table 3, Fig. 6A and B). Egger regression was used to provide statistical evidence of funnel plot symmetry, indicating that there was no significant publication bias (Table 3).

**Table 3:**
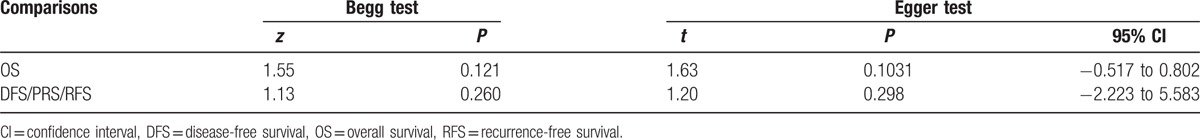
Publication bias of miR-218 for Begg test and Egger test.

## 4 Discussion

Recently, mounting evidence shows that miRNAs in cancer research has substantially changed the understanding of gene regulation, as an important cellular molecules involved in the normal and pathological states,^[31]^ miRNAs are important regulators of gene expression in tumor development by target genes and tumor suppressors or via directly exerting corresponding functions as oncogenes or tumor suppressors.^[32,33]^ In recent years, numerous studies have investigated that aberrantly expressed miRNAs in different types of cancer, they can be used as novel prognostic biomarkers of tumor.^[34–36]^

MiR-218 is a vertebrate-specific miRNA that has been predicted and experimentally confirmed to play a crucial role in tumorigenesis and tumor progression by regulating the expression of potential targets.^[37,38]^ MiR-218 have found to serve as a candidate tumor suppressor in targeting multiple cancer by regulation of relative gene expression.^[39,40]^ Mathew et al^[41]^ identified a miR-218-RTK-HIF2α signaling axis which promotes tumor angiogenesis and glioblastoma multiforme (GBM) cell survival, especially for necrotic mesenchymal tumors. Meanwhile, it was demonstrated that silencing of miRNA-21 promotes migration and invasion of breast cancer through Slit2-Robo1 pathway.^[42]^ Importantly, these results suggested that miR-218 acts as a potential tumor suppressor by targeting multiple cancer phenotype-associated genes in medulloblastoma, including RICTOR, CDK6, and cathepsin B (CTSB).^[43,44]^ However, significance of miR-218 expression with clinicopathological factors and/or prognosis of cancers are unclear.

In the present study, we conducted this analysis of the published literature to identify a group of miR-218 for which the data support validation as prognostic biomarkers of cancer outcomes. Due to the included studies used a variety of indices to evaluate tumor progression, such as DFS, PFS, and RFS, we combined these indices to evaluate the prognostic value of miR-218.

To the best of our knowledge, our meta-analysis is the first to critically examine available literature and identify the prognostic role of miR-218 in various cancers. The results demonstrated that expression of miR-218 was significantly correlated with OS (HR = 2.61, 95% CI: 2.11–3.22, *P* < 0.001) and DFS/PFS/RFS (HR = 2.73, 95% CI: 2.08–3.58, *P* < 0.001) in cancer, further demonstrating the predictive value of miR-218. Our stratified analysis suggested a closer relationship between rising miR-218 levels and poor survival in Asians and Caucasians. Among 10 studies reporting, four were related to DTC. Therefore, we performed a subgroup analysis of DTC. The result also revealed that reduced miR-218 yielded worse OS and DFS/PFS/RFS in DTC. Due to the lack of eligible studies reporting for each cancer type, further studies are required to determine whether pathological cancer types impact the prognostic role of miR-218.

Studies show that the main reason for the high mortality of cancer is the invasion and metastasis.^[44]^ Elevated expression of miR-218 inhibited the invasion and migration of cancer cells,^[45–47]^ it is currently believed that several types of deregulated miR-218 and its downregulation is associated with a poor prognosis.^[48]^ These results show that miR-218 play a tumor suppressor and decreased miR-218 expression in the tissue or serum was associated with OS and DFS/PFS/RFS. However, numerous published studies have been reported that miR-218 can regulate tumor invasion,^[49,50]^ the exact clinicopathologic significance and prognostic of miRNA-218 in cancers remain inconclusive.

Although meta-analysis is robust, our study also has several limitations that should be acknowledged. Firstly, the reliability of our results is questionable in light of the number of eligible studies for OS and DFS/RFS/RFS. Additionally, the patient populations were limited to Asia, and North America, lacking data from other regions, which might impact the statistical power of analysis, and ethnic bias might be possible, even though the statistical test did not show it. Secondly, the number of individual prognostic studies dealing with certain tumor type was not sufficient, which might impact the statistical power of analysis. Therefore, well-designed clinical studies with larger sample sizes should be carried out in the future. Thirdly, a clear definition should be made about the cutoff value of miR-218 level for outcomes. To date, most investigators use median or mean value in their studies as the cutoff value and the accurate value were different. Fourthly, due to not all survival data of the eligible studies were given directly, some data were extracted from survival curves. These calculated HRs with corresponding 95% CIs might be brought several tiny errors. Finally, although there was no significant evidence of publication bias in this analysis, cautions should be taken, and the tendency for journals to publish positive results could also make certain bias.

In summary, our data demonstrated that lower miR-218 expression is significantly associated with poorer OS and DFS/PFS/RFS and may be a novel prognostic biomarker in some cancer types, further multicenter prospective clinical studies are needed to determine the association between miR-218 and cancer prognosis.
